# Diversity of *Bacillus*-like organisms isolated from deep-sea hypersaline anoxic sediments

**DOI:** 10.1186/1746-1448-4-8

**Published:** 2008-06-09

**Authors:** Andrea M Sass, Boyd A McKew, Henrik Sass, Jörg Fichtel, Kenneth N Timmis, Terry J McGenity

**Affiliations:** 1 Department of Biological Sciences, University of Essex, Wivenhoe Park, Colchester CO4 3SQ, UK; 2 School of Biosciences, Cardiff University, Cardiff CF10 3YE, UK; 3 School of Earth Ocean & Planetary Science, Cardiff University, Cardiff CF10 3YE, UK; 4 Institute for Chemistry and Biology of the Marine Environment (ICBM), University of Oldenburg, D-26111 Oldenburg, Germany; 5 Helmholtz Center for Infection Research, Inhoffenstrasse 7, D-38124 Braunschweig, Germany; 6 Institute for Microbiology, Technical University Braunschweig, Germany

## Abstract

**Background:**

The deep-sea, hypersaline anoxic brine lakes in the Mediterranean are among the most extreme environments on earth, and in one of them, the MgCl_2_-rich Discovery basin, the presence of active microbes is equivocal. However, thriving microbial communities have been detected especially in the chemocline between deep seawater and three NaCl-rich brine lakes, l'Atalante, Bannock and Urania. By contrast, the microbiota of these brine-lake sediments remains largely unexplored.

**Results:**

Eighty nine isolates were obtained from the sediments of four deep-sea, hypersaline anoxic brine lakes in the Eastern Mediterranean Sea: l'Atalante, Bannock, Discovery and Urania basins. This culture collection was dominated by representatives of the genus *Bacillus *and close relatives (90% of all isolates) that were investigated further. Physiological characterization of representative strains revealed large versatility with respect to enzyme activities or substrate utilization. Two third of the isolates did not grow at *in-situ *salinities and were presumably present as endospores. This is supported by high numbers of endospores in Bannock, Discovery and Urania basins ranging from 3.8 × 10^5 ^to 1.2 × 10^6 ^g^-1 ^dw sediment. However, the remaining isolates were highly halotolerant growing at salinities of up to 30% NaCl. Some of the novel isolates affiliating with the genus *Pontibacillus *grew well under anoxic conditions in sulfidic medium by fermentation or anaerobic respiration using dimethylsulfoxide or trimethylamine *N-*oxide as electron acceptor.

**Conclusion:**

Some of the halophilic, facultatively anaerobic relatives of *Bacillus *appear well adapted to life in this hostile environment and suggest the presence of actively growing microbial communities in the NaCl-rich, deep-sea brine-lake sediments.

## Background

Numerous basins filled with highly saline, anoxic waters have been discovered on the seafloor of the Eastern Mediterranean Sea, the Red Sea and the Gulf of Mexico. Within the Eastern Mediterranean the hypersaline brine lakes are situated on the Mediterranean Ridge, a submarine mountain chain emerging from subduction processes at the collision zone of the African and European tectonic plates [1]. Tectonic processes have resulted in folded and deformed sediments leading to seawater coming into contact with evaporites that were deposited during the Messinian salinity crisis (5.96 to 5.33 million years ago) [2,3]. The outcropping salt is dissolved and the resulting highly saline brines can accumulate in depressions in the seafloor [1]. Owing to the weak currents at such a depth and the large difference in density, the hypersaline brines do not mix with the overlying seawater, forming an extremely steep chemocline at the interface with a vertical extension of about 2 meters [4]. The hypersaline brine lakes are unique and hostile environments, characterized by extremely high salt concentrations, anoxia with high sulfide concentrations, and the high pressure typical of a deep-sea environment [5].

**Figure 1:**
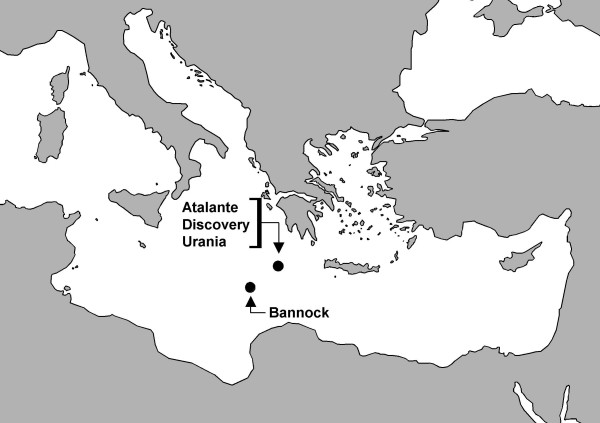
Location of the four deep-sea hypersaline anoxic basins within the Eastern Mediterranean Sea (coordinates for the basins: L'Atalante 35.18 N 21.41 E, Discovery 35.17 N 21.41 E, Urania 35.14 N 21.31 E, Bannock 34.17 N 20.00 E).

Four of the basins in the Eastern Mediterranean Sea, l'Atalante, Bannock, Discovery and Urania basins (Fig. 1) have been sampled and studied to better understand their biogeochemistry, ecology and biotechnological potential. The physico-chemical properties of the hypersaline brine lakes have been described by van der Wielen et al. [5], but in brief l'Atalante and Bannock brines contain ions roughly in proportion to those found in seawater but almost at the point of sodium chloride saturation (~8 times seawater concentration). The Urania brine has a slightly lower salinity, but exhibits one of the highest sulfide concentrations measured in marine environments [5,6]; it also has a very high methane concentration and in parts an elevated temperature, possibly caused by a deep source of the brine [7]. The Discovery basin is unique in that it derives from bischofite (MgCl_2_·6H_2_O), resulting in a ~5 Molar magnesium chloride brine [8], representing the marine environment with the lowest reported water activity [9].

Microbial activity within the basins has been demonstrated by hydrolytic enzyme activities, methanogenesis and sulfate reduction in all four basins [5], and depletion of ^34^S in sulfide, indicating microbial sulfate reduction in Urania Basin [6,10]. However, in the MgCl_2_-rich Discovery brine lake, Hallsworth et al. [9] did not detect messenger RNA coding for enzymes central to sulfate reduction and methanogenesis, and attributed this to the chaotropic nature of MgCl_2_. In contrast, the seawater – brine-lake interfaces for Bannock and l'Atalante basins have diverse and active microbial communities largely dominated by Bacteria [11,12]; and novel esterases that function at high pressure and over a wide range of salinities are indicative of microbes specifically adapted to the steep, deep-sea halocline between seawater and Urania brine lake [13].

In the sediments of l'Atalante brine-lake, viral abundance and virus to prokaryote abundance ratio are similar to those reported in oxic, deep-sea sediments [14]. The phospholipid-linked fatty acid (PLFA) compositions of the brine-lake sediments from Bannock, Urania and l'Atalante are similar to each other, and distinct from the PLFA composition of Discovery brine-lake sediment, which is more typical of deep-sea Mediterranean sediments outside of the hypersaline brines [15]. To date, a small number of cultured microorganisms has been reported, primarily from the seawater-brine lake interface of Bannock and Urania basins [6,11,16-18], but during these studies no isolates had been obtained from the brine-lake sediments. One of the aims of our study was to investigate the microbiota of the brine-lake sediments, and many isolates were obtained from sediments of all four basins, with the vast majority being spore-forming bacteria related to the genus *Bacillus*.

Members of the order *Bacillales *are found in almost every environment on earth from the stratosphere [19] to the deep subsurface [20-22]. This ubiquity is, in part, attributed to their ability to form resilient spores that can be transported over long distances [23], but a frequently overlooked feature of the order *Bacillales *is their great metabolic versatility and ability to grow under physico-chemical extremes. In this study we focus on the *Bacillus*-related isolates from the brine-lake sediments. Such deep-sea topographical depressions will collect all sorts of material, including bacterial spores, but here we provide evidence that some of the microbes from the brine-lake sediments have the potential to be active *in situ*.

## Results and Discussion

### Sediment characteristics

In contrast to the oxidized sediments in the vicinity of the basins, which had a beige-brown colour at the top, the anoxic sediments within the l'Atalante and Bannock basins were dark grey with a black surface layer approximately 1-cm thick, that was less compacted than the underlying layer and very easily disturbed. The top layer of the Discovery basin sediments was jet-black, and also not very compacted and very viscous owing to the high magnesium chloride content. There was no obvious layering of this sediment. The sediments of the Urania basin in contrast were of a light grey color. Gas bubbles developed in the Urania sediment shortly after retrieval of the multicorer, possibly due to out-gassing of methane or carbon dioxide under atmospheric pressure. The upper two centimeters of the anoxic sediments were nearly liquid, so they could only be sampled with a pipette and it was not necessary to mix the sediment with ambient water to form slurries before processing.

### Viable cell count

The highest colony-forming units (CFU) per ml of sediment were observed on artificial seawater, with estimated numbers of 1.1 × 10^4 ^for l'Atalante, 4.5 × 10^3 ^for Bannock, 1.3 × 10^3 ^for Urania and 5 × 10^2 ^for Discovery basins. The number of CFU was generally one order of magnitude lower on medium with elevated salinity (12%, medium B/2) than on artificial seawater. No growth was observed on medium B with 24% salinity. Total cell counts were not available from this study, but Sass [24] obtained a total cell count from Urania basin sediment of 3.6 × 10^7 ^cells ml^-1 ^which was similar to counts obtained by Danovaro et al. [14] for l'Atalante basin sediments (5.7 × 10^7 ^cells ml^-1^; converted using a density value of 1.23 g ml^-1 ^[5]), indicating that we cultivated about 0.004 to 0.02% of the microbes in the sediment.

The lowest counts were found in Discovery brine-lake sediment, and given that 5 M MgCl_2 _inhibits growth but preserves cell structure [9], it can be assumed that this provides a baseline for inactive but culturable microorganisms in the basin. This is supported by the findings of Polymenakou et al. [15] that PLFA profiles from sediments in the Discovery basin were similar to those from 'normal' hemipelagic Mediterranean sediments, suggesting that the majority of the source organisms for the PLFA were introduced into the basin by sedimentation or lateral flows. Sediments of the other three basins (l'Atalante, Bannock and Urania) had PLFA patterns that clearly differed from those of sediments outside the brine lakes revealing distinct and probably active communities [15]. Two questions emerge from these observations: were isolates similar between the four basins, and do they demonstrate the potential to be active *in situ*?

### Number and affiliation of isolates

A total of 89 strains were isolated from the sediment samples (29 from l'Atalante, 30 from Bannock, 16 from Discovery, and 14 from Urania basins). Screening by Amplified Ribosomal DNA Restriction Analysis (ARDRA) and partial sequencing of the 16S rRNA genes revealed that 80 strains (90% of those isolated) were related to the *Bacillales*. The other nine strains were related to *Halomonas aquamarina *(4 strains), *Pseudomonas *sp. (4 strains) and *Alteromonas *sp. (1 strain). Since *Alteromonas *and *Halomonas *spp. were previously shown to be dominant organisms in the chemocline but less abundant in the brine [6] we assume that they were not indigenous sediment bacteria but introduced by sedimentation. Spore-forming bacteria however were more typically isolated from brine-lake sediments, and only rarely from the chemocline (data not shown). Therefore, in the present study we focused on isolates affiliating with the *Bacillales*. Out of the 80 strains from the *Bacillales *25 representative strains with differing ARDRA profiles were selected for physiological characterization and detailed phylogenetic analysis. Of these 25 ARDRA groups, 17 were from one basin only, six were found in two basins, and two were found in three basins. There were no clear trends in the distribution of the isolates in the different basins; however, although numbers are relatively small, it is notable that one of the ARDRA groups represented by strain AS5, had 14 strains that were isolated only from l'Atalante and Bannock basins, the most geochemically similar, yet geographically distant basins.

**Figure 2:**
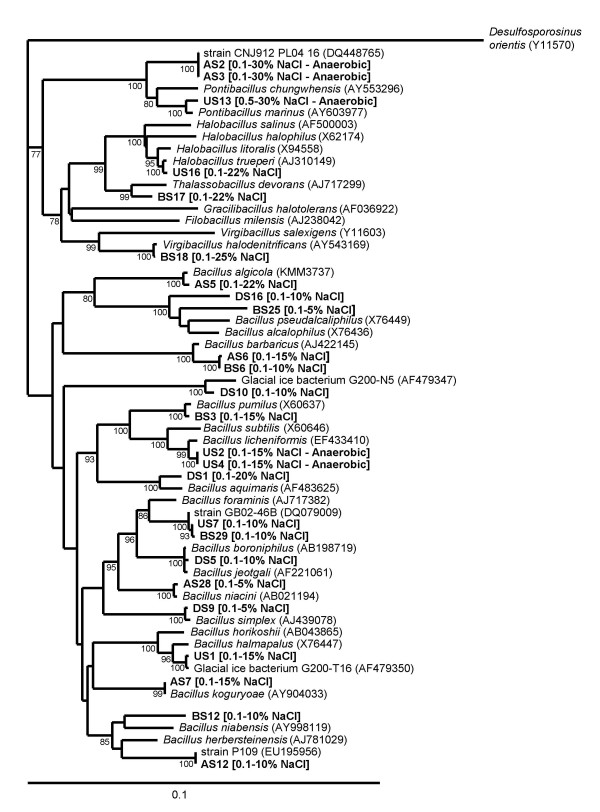
Phylogenetic tree based on 16S rRNA sequences of isolates from l'Atalante (AS), Bannock (BS), Discovery (DS) and Urania (US) brine-lake sediments. The tree was constructed based on an alignment of approximately 1340 base pairs from the isolates and their closest relatives. Uncharacterized strains were included in the tree when there was no closely related named species. For the named species, the sequence from the type strain was used. Accession numbers of reference strains are given in round brackets. The scale bar corresponds to 0.1 substitutions per nucleotide position. Figures (%) at branching points represent significance of branching order by bootstrap analysis (1000 replicates, bootstrap values above 75% are shown). For the strains isolated from the deep-sea brine-lake sediments, the NaCl range for growth and the ability to grow under anaerobic conditions are indicated in square brackets. Note that while strain US13 did not grow fermentatively, it grew using dimethylsulfoxide or trimethylamine *N-*oxide as electron acceptor.

Most of these *Bacillus*-like isolates are closely related to previously cultivated organisms (Fig. 2), many of which are moderately halophilic or alkaliphilic. Six strains (from l'Atalante, Urania and Bannock, but not from Discovery basin) belong to a cluster accommodating genera with many halotolerant representatives like *Halobacillus*, *Virgibacillus *and *Pontibacillus *(see Fig. 2) [25,26]. All of these strains were isolated on a medium with elevated salt concentration (12% NaCl).

### Phenotypic and physiological diversity of isolates

**Figure 3:**
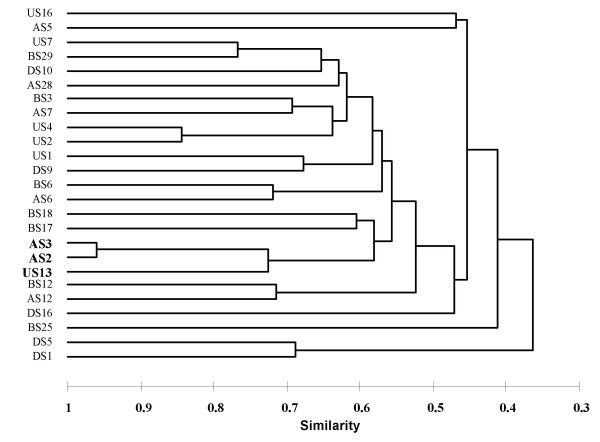
Dendrogram showing the phenotypic similarity of the isolates from l'Atalante (AS), Bannock (BS), Discovery (DS) and Urania (US) brine-lake sediments, based on 106 physiological and biochemical tests (see Methods). Similarities calculated with the AHC method, using the Jaccard coefficient and UPGA linkage. Clustering of the extremely halotolerant isolates AS2, AS3 and US13 (bold text) confirms their close phenotypic similarity in addition to their close phylogentic relationship (see Fig. 2).

The isolates were Gram-positive and, with the exception of strain BS3, formed endospores. All but four isolates were found to be motile by peritrichous flagella (Table 1). The pairs of strains that were phylogenetically most similar, such as AS2 and AS3, US2 and US4, US7 and BS29, and AS6 and BS6 (Fig. 2) were also phenotypically most similar (Fig. 3). The isolates were physiologically diverse (Table 1) particularly when considering their enzyme activities and substrate utilization capacities (Table 1). Generally, the isolates grew well on yeast extract, peptone, casamino acids and a range of carbohydrates and amino acids, while fatty acids or alcohols were less commonly used. A few strains grew also on aromatic compounds or *n-*alkanes. Compared with the other isolates, strains DS1 and DS5 had limited catabolic capabilities, growing only on five and three substrates, respectively. Interestingly, one of them (strain DS1) grew well on *n-*alkanes.

**Table 1:** Phenotypic characteristics of isolates from brine-lake sediments

	L'Atalante							Bannock							Discovery					Urania					
				
	AS2	AS3	AS5	AS6	AS7	AS12	AS28	BS3	BS6	BS12	BS17	BS18	BS25	BS29	DS1	DS5	DS9	DS10	DS16	US1	US2	US4	US7	US13	US16
Salinity of isolation medium [% NaCl]	12	12	12	3.5	3.5	3.5	3.5	3.5	3.5	3.5	12	12	3.5	3.5	12	3.5	3.5	3.5	3.5	3.5	3.5	3.5	3.5	12	12
Max. NaCl [%]	30	30	22	15	15	10	5	15	10	10	22	25	5	10	20	10	5	10	10	15	15	15	10	30	22
Growth at																									
4°C	-	-	-	-	-	+	-	-	-	-	-	-	-	-	+	+	+	+	-	+	-	-	-	-	-
45°C	+	+	-	+	+	-	+	+	+	-	+	+	-	+	+	+	+	+	-	+	+	+	+	+	-
53°C	-	-	-	-	-	-	-	+	-	-	-	-	-	-	-	-	-	-	-	-	+	+	-	-	-
pH 5.5	+	+	+	+	+	+	+	+	+	+	+	+	-	-	+	+	+	+	-	+	+	+	-	-	-
pH 6.5	+	+	+	+	+	+	+	+	+	+	+	+	-	+	+	+	+	+	-	+	+	+	+	-	+
pH 9.0	+	+	+	+	+	+	-	+	+	+	+	+	+	+	+	+	+	+	+	+	+	+	+	+	+
Fermentation^1)^	+	+	-	-	-	-	-	-	-	-	-	-	-	-	-	-	-	-	-	-	+	+	-	-	-
Motility	+	+	+	+	+	+	-	+	+	+	+	+	+	-	+	+	-	+	+	+	+	+	-	+	+
Enzyme activity																									
Polymer degr.^2)^	2	2	4	4	4	2	3	4	3	2	3	2	0	5	5	4	4	3	1	4	6	6	5	2	3
Nitrite prod.	-	-	-	-	-	+	+	-	-	-	-	+	+	+	+	+	-	-	-	-	+	+	+	-	+
β-Galactosidase	+	+	+	+	+	+	+	-	+	+	-	+	+	+	+	+	-	+	-	-	+	+	+	-	+
Arginine dehyd.	-	-	-	-	-	-	-	-	-	-	-	-	+	-	-	-	-	-	-	-	+	+	-	-	-
Urease	-	-	-	-	-	-	+	-	-	-	-	+	-	-	-	-	-	+	+	-	+	-	-	-	-
Acetoin prod.	-	-	-	+	+	+	+	+	+	+	+	+	+	+	+	+	+	+	+	+	+	+	+	+	-
Catalase	+	+	+	+	+	+	+	+	+	+	+	+	+	+	-	+	+	+	-	+	+	+	+	+	-
Oxidase	-	-	-	-	-	+	+	-	-	+	+	+	-	-	-	-	+	-	-	+	+	-	-	+	-
Substrate utilization^3)^																									
Poly- and disaccharides (8)	5	5	5	7	6	5	6	5	6	6	6	6	6	6	0	0	6	5	3	6	4	4	6	4	6
Monosaccharides (14)	9	9	5	6	8	9	8	9	5	9	10	9	6	8	3	1	12	13	11	10	12	12	9	9	10
Alcohols (5)	1	1	0	1	1	1	1	1	1	1	2	1	1	1	1	1	5	3	0	1	1	1	1	1	1
Fatty acids (4)	0	0	0	0	1	0	1	0	0	0	2	1	0	1	0	0	2	1	0	2	0	0	4	0	0
Carboxylic acids (7)	5	5	0	5	6	0	3	4	5	2	6	7	0	7	1	1	7	7	6	4	6	4	7	6	0
Amino acids (20)	7	7	0	8	6	0	4	5	3	2	9	0	7	3	0	0	5	8	5	7	4	4	6	4	7
Betaine, Salicylate, benzoate	0	0	0	0	0	0	0	0	0	0	2	2	0	0	0	0	0	1	0	0	0	0	0	0	1
Alkanes (2)	0	0	0	0	0	0	0	0	0	0	0	0	2	0	2	0	0	0	0	0	0	0	0	0	0

^1) ^Fermentation: fermentative growth with yeast extract. Note that while strain US13 did not grow by fermentation, it grew using dimethylsulfoxide or trimethylamine N-oxide as electron acceptor.

^2) ^Number of positive results for polymer hydrolysis out of seven different tests; all strains used the complex substrates yeast extract, casamino acids and bactopeptone; all strains tested positive for tryptophan deaminase; and all strains tested negative for N_2 _production from nitrate, lysine decarboxylase, ornithine decarboxylase, H_2_S production, indole production. Degr. = degradation, prod. = production, dehyd. = dehydrogenase.

^3) ^Numbers in brackets indicate the number of tested compounds in the respective category (see Methods for details of substrates tested); none of the isolates used serine, leucine, methionine or tyrosine. All isolates grew with yeast extract, peptone and casamino acids.

All strains were mesophilic, growing between 12 and 37°C, indicating that they could grow within the temperature range experienced at the surface of the Mediterranean and the deep-sea, including the brine lakes which have a temperature of 13.9 to 16.7°C [5]. Some isolates also grew at 4°C, whereas others grew at 45 or even 53°C (Table 1). With the exception of strain US13 (minimum salinity for growth of 0.5% NaCl), all strains grew in media containing only 0.1% NaCl, and most had a growth optimum between 1 and 5% NaCl, suggesting that they were marine and incapable of growth in the brine-lake sediments. None of the five isolates from Discovery brine-lake sediment grew in media with an MgCl_2 _concentration exceeding 7.5%, and therefore were not adapted to *in-situ *conditions. However, the eight strains isolated on medium B/2 with 12% NaCl were generally the most halotolerant (Table 1). Three of these strains (AS2, AS3 and US13) were capable of growing at extremely high salt concentrations, similar to those in the basins. They grew up to 30% NaCl with an optimum of 10–15% NaCl. Strains AS2 and AS3 also grew by fermentation in anoxic artificial seawater as well as in medium with 20% NaCl (Table 1), and all three strains (AS2, AS3 and US13) could use dimethylsulfoxide and trimethylamine *N-*oxide as terminal electron acceptors (data not shown).

### Are the brine-lake sediment communities dominated by inactive endospores?

The high proportion of *Bacillus*-related strains among the isolates in this study, although no pasteurisation was performed to select for spore formers, suggests that many the *Bacillus-*like isolates might originate from dormant endospores and that most of the non-spore-forming marine microorganisms that might drift into the brine do not survive. This is supported by the general lack of correlation between the affiliation of the isolates and the basin of origin, and especially by failure of many strains to grow at *in-situ *salinities. Endospores can be stained by the commonly used dyes like acridine orange and DAPI [27], and so can be expected to contribute to the total cell counts. Endospore numbers were estimated from dipicolinic acid (DPA) contents [28] in sediment samples from the Urania (3.8 × 10^5 ^g^-1 ^dw sediment), Bannock (9.0 × 10^5 ^g^-1 ^dw sediment) and Discovery (1.2 × 10^6 ^g^-1 ^dw sediment) basins. These numbers are lower than those obtained from highly active coastal sediments where endospores were estimated to represent up to 3% of the total cell counts [28]. However, considering the low total cell counts in the range of 2.6 and 5.7 × 10^7 ^cells ml^-1 ^(note the different unit) in the brine-lake sediments it is apparent that the contribution of endospores to the brine-lake sediment microbial communities is in the same range (up to 5% of the total cell counts).

The capacity to form endospores may explain why *Bacillus*-like organisms can be isolated from almost all types of environment, e.g. soils, sediments, foods and water [29], including extreme environments like solar salterns [30,31], deep-sea hydrothermal vents [32] or brine-lake sediments (this study). Whether they play an important ecological role in most of these environments remains a matter of debate. In studies using a combination of culture-dependent and -independent methods members of the *Bacillales *are often found among the isolates, but rarely in DNA-based detection methods [33,34], suggesting that spore-forming bacteria are easily cultivated with standard methods although they represent only a small proportion of the microbial community. This might be explained by the fact that bacteria in most environments are strongly energy-limited, and it was shown that starved cells can be severely harmed by a substrate shock after exposure to high substrate concentrations as found in many standard media [35]. In contrast, endospores do contain enough energy for germination and are specifically adapted to quickly respond to substrate availability and by forming a vegetative cell they are able to proliferate. Therefore, it can be expected that they can easily outgrow other organisms after transfer onto microbiological media. On the other hand, endospores possess a very rigid and resistant cell wall and it is unclear to what extent endospores are successfully lysed and extracted using standard nucleic-acid extraction procedures [e.g. [36]]. The spores themselves may have an important ecological role, especially as they contribute to such a high proportion of total counts in sediments. For example, spores have been shown to catalyse the oxidation of Mn^2+ ^[37].

Spores of *Bacillus*-like organisms can rest dormant for long time periods and they are resistant to damage through desiccation and radiation for example [23]. *Bacillus*-like organisms have been isolated from deep subsurface sediments [21,22] and from inclusions inside materials like amber [38], salt crystals [39] or glacial ice [40,41] where the spores must have been included since the time of deposition. The age of the inclusions in the salt crystals and amber was estimated in the range of several million years [38,39], the age of those in glacial ice in the range of 5 to 750,000 years [40,41].

Therefore, the spores in the basin sediments may have drifted into the brines from environments far from the deep-sea hypersaline anoxic basins, accumulated in the brine-lake sediments and remained viable. The high salinity within the brine lakes could even have enhanced the preservation of the spores [9,14,42,43]. Alternatively it can be envisaged that they originate from the evaporites, as viable spores were reported from salt deposits tens of millions of years old [39], far older than the Messinian evaporates beneath the Mediterranean Sea.

### Evidence for active members of the *Bacillales *in brine-lake sediments

About two thirds of the strains tested did not grow under *in-situ *salinities and probably derived from dormant spores, whereas seven strains were very halotolerant and grew at salinities close to *in-situ *values. However, in the fermentation assay only two of these (strains AS2 and AS3) formed cultures turbid enough for photometric analysis. This is not unusual; many anaerobic bacteria form only low biomass. This can be explained by the low energy yield available from many fermentations. Otherwise many bacteria may rely on the activity of a syntrophic partner which is lacking when grown in pure cultures. An example of such bacteria are strains related to the genus *Rhizobium *isolated under anoxic conditions from Mediterranean sediments [44]. None of these formed turbid cultures under anoxic conditions. *Rhizobium*-related organisms were unexpected for deep-sea sediments, but were proven to be abundant members of the sediment microbial communities by quantitative PCR [45]. Whether the same is true for the *Bacillus*-like organisms in the brine-lake sediments needs to be investigated further. In any case, our data confirm that it is possible to isolate *Bacillus*-like strains that are physiologically capable of growing under the conditions in the brine, i.e. extremely high salinity combined with anoxia and a sulfide concentration of several millimol per liter.

## Conclusion

The topography of deep-sea hypersaline anoxic basins makes them excellent collecting bowls for material sinking from the surface and slumping from surrounding sediments. Most of the organisms entering the brine lake will be unable to tolerate the extreme salinity or high sulfide concentrations and so will die and release their cellular contents. This, together with the combined preservation effects of anoxia and low water activity, explains why l'Atalante brine-lake sediment has the highest concentration of extracellular DNA reported in a natural environment [14]. Spores, however, are better equipped to survive environmental shocks, explaining why we isolated such a high proportion of endospore-forming *Bacillus*-like organisms from the brine-lake sediments. Discovery brine lake, an almost saturated 5 M MgCl_2 _brine, is the most chaotropic large-scale environment on earth, and has the lowest water activity of any marine environment [9]. There is, however, conflicting evidence of microbial activity [5,9] in Discovery hypersaline brine. Several lines of evidence suggest that microbial activity is not favored in Discovery basin: undetectable mRNA from sulfate-reducing bacteria and methanogens compared with the upper part of the chemocline and l'Atalante basin [9], similarity of PLFA profiles with deep-sea sediments outside of the hypersaline brine [15], and the high redox potential (10 mV) compared with the three other brine-lake sediments (-82 to -136 mV) [15]. Here we provide evidence that Discovery Basin sediments have the lowest viable counts yet the highest endospore counts; also this, and a previous study [9], have shown that microbes could not be isolated, nor sub-cultured on media with MgCl_2 _concentrations approaching those found in Discovery brine. This suggests that a large proportion of the microbes in Discovery sediment are inactive, but leaves open the possibility of finding novel extremophiles specifically adapted to high concentrations of this extremely chaotropic salt.

We provide the first report of isolates from the deep-sea hypersaline brine-lake sediments, and while most did not have the potential to be active *in situ*, some isolates, clustering within the genus *Pontibacillus*, could grow at *in-situ *salinities and under anoxic conditions, either by respiration with dimethylsulfoxide or trimethylamine *N-*oxide as electron acceptor (AS2, AS3, US13) or by fermentation (AS2, AS3). This confirms that the NaCl-dominated brine-lake sediments contain active microbial populations as demonstrated in l'Atalante basin by leucine incorporation [14], and also supports the notion that the extremely high sulfide concentration in Urania brine lake [5,6], known to inhibit even sulfate-reducing bacteria [46], allows microbial processes to continue. This study raises tantalizing questions about the origin, distribution, survival and metabolic activity of vegetative microbial cells and endospores in some of the most extreme environments on earth: why for example were 14 isolates with identical ARDRA profiles found only in Bannock and l'Atalante brine-lake sediments, the most geochemically similar, but geographically remote basins? Answers will come from further analysis of isolates to obtain a detailed understanding of their physiology, as well as nucleic-acid based studies of microbial communities and endospores in, around and above the brine-lake sediments as well inside Messinian rock salt.

## Methods

### Site description and sampling procedures

Four different brine lakes in the Eastern Mediterranean were sampled between 20^th ^and 31^st ^August 2001 on a cruise with the R/V Urania. Three of them, l'Atalante, Discovery and Urania basin, are situated very close to each other on the north-eastern edge of the Mediterranean Ridge [47], only a few kilometers apart, whereas the Bannock basin is located on the southern edge of the ridge [48] (Fig. 1). Samples from the upper two centimeters of the sediments were taken with a box corer (Discovery basin) or a multicorer (all other basins). Immediately after retrieval, the sediment was aseptically transferred into serum bottles. The headspace was flushed with oxygen-free nitrogen and sealed by means of butyl rubber stoppers. Until processing, the samples were stored in the dark at room temperature. Dilution series from the sediment samples were made with a sterile solution of 25% NaCl.

### Enrichment, isolation and cultivation

For enrichment and isolation of bacteria six different oxic media were used, representing a combination of three different salt concentrations and two mixtures of organic substrates. Medium B reflected the salt composition of the Bannock basin [48]; the salinity was approximately 24%. It contained as major salts (in g l^-1^): NaCl 181.2, MgCl·6H_2_O 61, KCl 7.5, CaCl_2_·2H_2_O 2.2, Na_2_SO_4 _14.2, MgBr_2_·6H_2_O 1.2, H_3_BO_3 _0.3, TRIS 2.4. Minor constituents were added from stock solutions (in mM final concentration): SrCl_2 _0.05, LiCl 0.075, NaF 0.07, NH_4_Cl 0.5, KH_2_PO_4 _0.1. One ml of a trace element solution (SL10, [49]) was added and the pH adjusted to 7.6 with HCl. A solution of 10 vitamins [50] was added after autoclaving. Medium B/2 resembled medium B, but contained only half of the amount of the major salts. Medium SW consisted of artificial seawater which contained (in g l^-1^): NaCl 24.3, MgCl_2_·6H_2_O 10, KCl 0.75, CaCl_2_·2H_2_O 1.5, Na_2_SO_4 _4. The minor constituents, buffer and pH of the artificial seawater were as described above, additionally MgBr_2 _and H_3_BO_3 _were added from stock solutions to a final concentration of 0.4 mmol l^-1 ^each.

The organic substrates are denoted as CPS (casamino acids 0.5 g l^-1^, bactopeptone 0.5 g l^-1^, soluble starch 0.5 g l^-1^, glycerol 13 mmol l^-1^) or as M, a mixture of the following substrates (in 1 mM final concentrations, pH 7): glucose, glycerol, sodium acetate, sodium lactate, sodium fumarate, sodium succinate, alanine, glycine, sodium glutamate and sodium thiosulfate.

After six weeks of incubation at 20°C colonies from each positive plate were selected after their appearance, and isolated by streaking out repeatedly on the respective medium. Isolated strains were maintained on CPS-SW at 20°C. The number of CFU ml^-1 ^sediment was estimated from the mean number of colonies on the seawater-based medium with organic components CPS and M, using the calculation of Cavalli-Sforza [51].

### Phenotypic characterization

Tests for the presence of oxidase and catalase, Gram-staining and staining of flagella and spores were performed according to standard procedures [52]. For further enzyme tests the API 20 E-system (bioMérieux, Marcy L'Étoile, France; [53]) was used following the manufacturer's instructions. The degradation of Tween 80 and the following polymers was determined on CPS-SW agar (without starch) at 30°C, unless indicated otherwise. All results were confirmed with two independent replicates of each test. Amylase activity was detected by zones of clearing around colonies on medium with 2 g l^-1 ^of soluble starch (BDH Laboratories) after flooding plates with Lugol's iodine solution. TWEEN 80 esterase activity was detected by formation of precipitate around colonies on medium containing 1% w/v TWEEN 80 (BDH Laboratories). Gelatin liquefaction was tested by adding 12% w/v gelatin (Sigma^®^, Type A; 300 Bloom) instead of agar to CPS-T-SW (excluding starch); the solidified medium was inoculated with a needle and incubated at room temperature. Casein hydrolysis was demonstrated by zones of clearing around colonies on media to which 3 g l^-1 ^of sterile skimmed milk (autoclaved separately at 110°C for 40 min) had been added. Cellulase activity was detected by a zone of clearing around colonies after flooding plates containing 1% w/v low-viscosity carboxymethyl cellulose (Sigma) with 1 g l^-1 ^of Congo Red (Sigma). Ground chitin (50 g) from crab carapaces was dissolved by stirring for one hour in 2.5 l of 32% HCl. The solution was filtered through a cloth before adding sufficient deionised water (approximately 20 l) to precipitate the chitin. The precipitate was washed by centrifugation, until a neutral pH was obtained. The prepared chitin (0.5 g l^-1^) was added to CPS-T-SW (excluding starch) agar, and added as an overlay medium on top of standard CPS-T-SW, which was inoculated and incubated at 30°C. Chitinase activity was detected by a hydrolytic zone appearing as clear halos around colonies. DNAse activity was detected by zones of clearing around colonies growing on DNase Test Agar (Merck) with added NaCl (19.4 g l^-1^) after flooding plates with 1 M HCl.

Substrate utilization was tested in microtiter plates using liquid SW medium and the following compounds as sole carbon and energy source (final concentrations in brackets): (1) The poly- and disaccharides dextran, alginic acid, laminarin (each at 2 g l^-1^), cellobiose, sucrose, maltose, lactose, and trehalose (each at 5 mM), (2) the monosaccharides and their derivates glucose, fructose, galactose, mannose, arabinose, ribose, xylose, rhamnose, N-acetyl-glucosamine, glucosamine, gluconic acid, sorbitol, mannitol (each at 10 mM) and meso-erythritol (20 mM), (3) the alcohols glycerol (20 mM), methanol (20 mM), ethanol (20 mM), *n*-propanol (10 mM) and *n*-butanol (10 mM), (4) the fatty acids formate (20 mM), acetate (20 mM), propionate (10 mM) and butyrate (10 mM), (5) the carboxylic acids lactate, pyruvate, malate, succinate, fumarate, citrate (each at 20 mM) and α-ketoglutarate (10 mM), (6) the 20 common amino acids glycine, alanine, cysteine, proline, serine, threonine, valine (at 20 mM each), arginine, asparagine, aspartic acid, glutamine, glutamic acid, histidine, isoleucine, leucine, lysine, methionine, phenylalanine, tryptophan and tyrosine (at 10 mM each), (7) miscellaneous compounds: betaine (20 mM), benzoate (10 mM) and salicylate (10 mM), (8) the complex substrates yeast extract, casamino acids and bactopeptone (each at 1 g l^-1^), and (9) the *n-*alkanes, *n*-dodecane and *n*-hexadecane (1% v/v) as sole carbon and energy source were tested in oxic SW medium in sealed serum bottles. Growth was assessed visually after two weeks incubation at 30°C.

The range for growth at different temperatures, salt concentrations and pH values was tested in liquid CPS-SW medium in glass test tubes. The medium was amended with yeast extract (1 g l^-1^), and, below pH 7.0, PIPES (10 mM) was used as a buffer instead of TRIS. The salt concentrations tested ranged from 0.1 to 30% w/v NaCl, with the other major salts at a concentration of 0.5 mM. Growth was measured by turbidity at 430 nm.

Anaerobic growth was tested in screw cap bottles using liquid anoxic CPS-SW medium buffered with NaHCO_3 _(30 mM, autoclaved separately) replacing TRIS, Na_2_S (2 mM, autoclaved separately at 109°C) as reducing agent, resazurin (0.25 mg l^-1^) as redox indicator, and filter-sterilized glucose (4.5 g l^-1^) replacing starch.

### Cluster analysis

The results of the 106 phenotypic tests were coded for numerical analysis in binary format and a similarity dendrogram was drawn using the XLSTAT™ software package (version 2008.1.02; Addinsoft, Paris, France) using the Agglomerative Hierarchical Clustering (AHC) method, calculated with the Jaccard coefficient and UPGA (unweighted pair-group average) linkage.

### Screening for different 16S rRNA phylotypes by Amplified Ribosomal DNA Restriction Analysis (ARDRA)

Freshly grown colonies were suspended in 10 mM TRIS, pH 8, centrifuged and washed. The cells were then lysed by five freeze-thaw cycles (1 min in liquid nitrogen, followed by 5 min at 60°C). The lysate (0.5 – 1 μl) was used directly in the PCR. The 16S rRNA genes were amplified in a Gene Amp PCR System (Applied Biosystems, Foster City, CA, USA) using 0.05 units·μl^-1 ^final concentration of TaqPol DNA polymerase (Qiagen, Crawley, UK). The primers used were 27F (5'-AGA GTT TGA TCM TGG CTC AG-3') and 1492R (5'-TAC GGY TAC CTT GTT ACG ACT T-3') with an annealing temperature of 55°C. The PCR product was purified with the QIAquick system (Qiagen, Crawley, UK) and approximately 100 ng of the cleaned product was digested for 2.5 h at 37°C with the restriction enzymes *Alu *I (1 unit·μl^-1 ^final concentration, Roche Diagnostics, Mannheim, Germany). The resulting DNA fingerprints were analyzed after electrophoresis in a 3% w/v agarose gel (1 h, 100 V, 100 base pair ladder as standard) using a digital image analyzer and the Quantity One software package. One strain of each fingerprint-type was selected for partial sequencing of the 16S rRNA genes, and 25 strains with different ARDRA profiles and related to *Bacillaceae *were selected for phenotypic characterization.

### 16S rRNA gene sequencing and phylogenetic analysis

Purified PCR products were subjected to sequence analysis using the ABI PRISM Big Dye Terminator Cycle Sequencing kit version 2.0 and an ABI 310 automatic sequencer (Applied Biosystems, Foster City, CA, USA). The primers used to obtain sequence fragments were 518R (5'-CGT ATT ACC GCG GCT GCT GG-3'), 338R (5'-CTG CTG CCT CCC GTA GGA GT-3'), 357F (5'-ACT CCT ACG GGA GGC AGC AG-3'), 907R (5'-CCG TCA ATT CMT TTR AGT TT-3'), 1389R (5'-ACG GGC GGT GTG TAC AAG-3'). Sequence fragments were checked using the Chromas 1.45 software package [54] and contigs assembled using BioEdit 5.0.9 [55]. Assembled 16S rRNA sequences were compared to the European Bioinformatics Institutes database by online FastA searches [56]. Multiple sequence alignment of the isolates and their closest relatives was performed using CLUSTAL W [57] and GeneDoc Multiple Sequence Alignment Editor version 2.6.002 [58]. Phylogenies were constructed based on an alignment of approximately 1340 bp, with the PHYLIP software package [59], using the Jukes and Cantor [60] model of nucleotide substitution, and the neighbour-joining algorithm. Significance of branching order was determined by bootstrap analysis with 1000 resampled data sets. Sequences were deposited in the EMBL databases with accession numbers AM950291 to AM950315.

### Determination of dipicolinic acid (DPA) and estimation of endospore numbers

Numbers of endspores in sediment samples were estimated after extraction and analysis of dipicolinic acid (DPA), an endospore-specific biomarker [28]. Extraction and analysis were performed after Fichtel et al. [61]. In brief, 1.0 g of freeze-dried sediment was weighed into autoclavable 15-ml polypropylene tubes with screw caps. Duplicates were prepared for each sample to determine recovery: One of the sediment aliquots was suspended in 2.5 ml of sodium bisulfate buffer (50 mmol l^-1^, pH 1.2), the other one was spiked by suspending in 2.5 ml of buffer with 250 nmol DPA l^-1^. Both duplicates were autoclaved to completely release DPA from the endospores within the sediment. After cooling, the samples were centrifuged (4000 *g*, 5 min, 15°C), and the supernatants were filtered through cellulose acetate syringe filters (0.2 μm pore size, Nalgene Nunc International, Rochester, NY) into polypropylene vials. DPA concentrations were determined as Tb-dipicolinate complexes via HPLC with post-column complexation and fluorescence detection as described by Fichtel et al. [61]. Endospore numbers were estimated using an average cellular DPA content of 2.24 × 10^-16 ^mol per spore [28].

## Competing interests

The authors declare that they have no competing interests.

## Authors' contributions

TJM, KNT and AMS conceived the study, AMS sampled and obtained isolates, AMS and BAM performed most of the experimental work, JF performed DPA analysis. All were involved in developing the study and in the writing.
